# Foreign Gene Transfer in Termite Cells Using a Recombinant Vesicular Stomatitis Virus

**DOI:** 10.1673/031.008.5201

**Published:** 2008-09-18

**Authors:** Katharine L. Modisett, Christy D. Robinson, Ashok K. Raina, Alan R. Lax, Scott F. Michael, Sharon lsern

**Affiliations:** ^1^ Department of Cell and Molecular Biology, Tulane University, New Orleans, Louisiana 70118, USA; ^2^ Department of Tropical Medicine, Tulane University, New Orleans, Louisiana, 70112, USA; ^3^ Formosan Subterranean Termite Research Unit, USDA, ARS, New Orleans, Louisiana, 70124, USA; ^4^ Biotechnology Research Group and Department of Biological Sciences, Florida Gulf Coast University, Fort Myers, Florida 33965, USA

**Keywords:** termite, gene transfer, vesicular stomatitis virus, primary cell culture, cryopreservation

## Abstract

The Formosan subterranean termite, *Coptotermes formosanus* Shiraki, and the eastern subterranean termite, *Reticulitermes flavipes* (Kollar) (Isoptera: Rhinotermitidae), are well known for their destruction of human dwellings and flora in the tropics and subtropics. A method to deliver foreign genes into termite cell cultures would provide a controlled environment to facilitate the study of key regulatory functions at the molecular and cellular level. Here a method for the establishment and cryopreservation of primary embryonic termite cell cultures is described. Evidence is presented of viral-mediated gene transfer in these cells and foreign gene expression using a recombinant vesicular stomatitis virus vector.

## Introduction

Termites have been the objects of study for over a century due to their ability to destroy cellulose material, their symbiotic and parasitic associations with various microorganisms including fungi, bacteria, and protists, their complex social life, and their cryptic behaviors (Kofoid 1946). In the United States, the subterranean termites (Isoptera: Rhinotermitidae) which include the Formosan subterranean termite, *Coptotermes formosanus* Shiraki, and the native eastern subterranean termite, *Reticulitermes flavipes* (Kollar), are important urban pests, destroying wooden structures and living trees. *C. formosanus*, originally from South East Asia, was introduced into Honolulu, Hawaii via commerce about 1900 and later into Texas and Louisiana in the mid 1960s (Gay 1969). Quarantines to prevent spread proved ineffective due to lack of enforcement and the cryptic nature of termite infestations. *C. formosanus* infestations have now been found as far north as North Carolina and it is expected that only climatic factors such as temperature and humidity will limit their range in North America (Lax and Osbrink 2003). A mature colony of *C. formosanus* produces annual swarms of winged alates that mate and establish new colonies (Raina et al. 2003). *C. formosanus* colonies can have up to several million foraging workers, and due to their large colony size can cause more damage than the native species, *R. flavipes*.

Since in some urban settings the integrity of many wooden structures and dwellings of historical and economic importance has been compromised by *C. formosanus* infestation, there is a need to develop alternative termite management strategies (Lax and Osbrink 2003). Preventive treatment and damage repair are estimated to cost over $1 billion annually in the United States. Though effective, long-lasting organochlorine and organophosphate termicides have been taken off the market due to health and environmental concerns. Newer soil barrier termiticides have been found to have lower toxicity to mammals and reduced environmental impact, but have been known to fail in controlling structural infestations. In addition to chemical pesticides, monitored baiting technologies have been developed. These technologies are more environmentally friendly as they use reduced amounts of toxin. They are slower acting and rely on foraging termites to deliver the toxin to the nest with the potential of eradicating the entire colony. Current research efforts have focused on the use of biological control agents using natural pathogens such as fungi, bacteria, nematodes, and viruses, but much work remains to be done.

A better understanding of the basic biology of termites at the molecular and cellular level could provide novel insights for the development of improved management and control strategies of *C. formosanus* populations by revealing new insecticide targets. In order to accomplish this, cellular and molecular tools, which include methods for termite cell culture and gene delivery and expression in termite cells, will be required. To facilitate these studies, we have developed methods for the culture and cryop-reservation of primary embryonic termite cells and the infection of these cultures, as well as adult termite cells, with a recombinant virus, vesicular stomatitis virus (VSV), resulting in expression of a foreign transgene.

VSV, a negative-stranded RNA rhabdovirus of the genus *Vesiculovirus*, enters target cells via receptor-mediated endocytosis. Viral cores containing the viral genome and replication machinery are released into the cytoplasm following fusion mediated by the viral surface glycoprotein, the G protein (Matlin et al. 1982). We hypothesized that VSV might be a useful gene transfer vector in termites due to its broad host range and the occurrence of natural VSV infections in a variety of insect species and vertebrate hosts (Gillies and Stollar 1980; Shope and Tesh 1987; Tesh et al. 1970; Tesh et al. 1972). It has been recently shown that a recombinant VSV vector can experimentally infect diverse invertebrate organisms such as the freshwater microcrustacean, *Daphnia* and the nematode, *Caenorhabditis elegans* (Robinson et al. 2006; Schott et al. 2005). The use of these tools could provide insight into the basic biology of termites. In addition, these results provide evidence of viral infection in termites, which may lead to viral pathogen-mediated control strategies.

## Materials and Methods

### Termites

Eggs and workers from the invasive Formosan subterranean termite, *C. formosanus*, were collected from laboratory colonies at the Formosan Subterranean Termite Research Unit, USDA, ARS, New Orleans, LA. Eggs from the native eastern subterranean termite, *R. flavipes*, were collected from field sites in New Orleans, LA.

### Primary embryonic termite cell cultures

Approximately 230 *C. formosanus* or *R. flavipes* eggs with advanced stage embryos were collected in a microfuge tube containing sterile de-ionized H_2_O (dH_2_O). Eggs were surfaced sterilized by sequential washing with 1 ml of the following solutions: 0.05% (w/v) NaHClO_4_ (x1), dH_2_O (x2), 70% (v/v) ethanol (x1), twice in dH_2_O, and twice in termite cell culture medium (TCCM) (80% (v/v) Schneider's Drosophila Medium (Invitrogen Corporation, www.invitrogen.com) in dH_2_O, pH 7.0 with 10% (v/v) heat-inactivated fetal bovine serum , 10 µg/ml gentamicin, 100 units/ml penicillin, 100 µg/ml streptomycin, and 2.5 µg/ml amphotericin B). For each wash, the microfuge tube was inverted 15 times, eggs were allowed to settle to the bottom and solution was removed without disturbing the eggs. Eggs were then resuspended in 500 ul TCCM, and embryos were disrupted and removed from eggs by gentle homogenisation using a sterile disposable tissue homogenizer micropestle (Fisher Scientific, www.fishersci.com). Embryonic tissues were collected by centrifugation at 201xg for 5 minutes at 4°C, resuspended in TCCM, and cultured at 27°C. Cell viability was analyzed with nucleic acid stains SYBR 14 and propidium iodide (Molecular Probes, www.probes.com) using fluorescence microscopy (Garner et al. 1994). Viability depended on the degree of homogenisation. Best results were observed when eggs were crushed twice with the micropestle, followed by inversion of the microfuge tube several times, and then crushed twice more with the micropestle.

### Cryopreservation of embryonic termite cell cultures

After gentle homogenisation, primary embryonic termite cell cultures in 500 µl of TCCM were centrifuged at 201xg for 5 minutes at 4°C. Embryonic termite cell cultures were resuspended in 1.5 ml of cryopreservation media (5% (v/v) dimethyl sulfoxide in fetal bovine serum), transferred to a 2.0 ml cryovial (Nalgene Nunc International, www.nalgenunc.com), immediately placed in a room temperature Cryo 1°C freezing container which achieves a cooling rate of -1°C per min (Nalgene), and incubated at -80°C overnight. For long term storage, embryonic termite cell cultures were transferred to a liquid nitrogen storage tank within 24 h. Viability of cryo-preserved cultures was determined using nucleic acid stains SYBR 14 and propidium iodide (Molecular Probes,) using fluorescence microscopy (Garner et al. 1994).

### Propagation of VSV-GFP

An infectious cDNA clone of VSV was engineered to contain the jellyfish green fluorescent protein (GFP) open reading frame inserted at the leader-N gene junction. (Cherry et al. 2005; Whelan et al. 1995; Whelan et al. 2000). VSV-GFP was propagated in baby hamster kidney cells (BHK-21) (American Type Culture Collection, www.atcc.org) in Dulbecco's Modified Eagle Medium (Invitrogen) supplemented with 10% (v/v) fetal bovine serum, 2mM L-alanyl-L-glutamine (Glutamax, Invitrogen), 100 units/ml penicillin, and 100 µg/ml streptomycin. Initial infection was done with a multiplicity of infection of 0.01 in serum-free medium for 1 h at 37°C. Eighteen hours post-infection, supernatants were collected, clarified by centrifugation at 3,220 × g for 5 min at 4°C, and filtered through a 0.45 micron filter (Whatman). Virus was concentrated by centrifugation using an SW28 rotor at 72,000 × g for 90 min at 4°C. Virus-containing pellets were resuspended in phosphate-buffered saline (PBS) pH 7.2 overnight on ice and stored at -80°C. Tenfold serial dilutions of virus were titered on BHK-21 cell monolayers. Fluorescent foci were counted and virus titers were expressed as focus-forming units (FFU) per ml.

### Infection of embryonic termite cultures with recombinant VSV

Primary embryonic *C. formosanus* and *R. flavipes* cells were infected with 1.5 × 10^7^ FFU of VSV-GFP in 500 µl TCCM in a well of a 24-well tissue culture plate and cultured at 27°C. GFP expression was analyzed by fluorescence microscopy and immunoblot assay.

### Immunoblot of VSV-GFP infected embryonic termite cultures

VSV-GFP infected embryonic termite cells were collected and lysed in 20 µl of cell lysis buffer (1% (v/v) Triton X-100, 20 mM Tris pH 7.5). Twenty µl of sample cell lysate was added to 5 µl of sample loading buffer (35 mM Tris-HCl pH 6.8, 2.8% (w/v) SDS, 20% (v/v) glycerol, 200 mM dithiothreitol, and 0.001% (w/v) bromophenol blue). Samples were heated to 100°C, separated by SDS-PAGE in a 10% Tris-HCl gel (Bio-Rad, www.biorad.com), and transferred by electroblotting onto a PVDF membrane (Millipore, www.waters.com) in 25 mM Tris base, 192 mM glycine, and 20% (v/v) methanol. Blots were probed overnight at 4°C with a rabbit anti-GFP antibody (BD Living Colors A.v. Peptide Antibody; BD Biosciences, Clontech, www.clontech.com) diluted 1:100 in 5% nonfat dry milk in PBS-Tween (1% (v/v) Tween-20 in PBS). Horseradish peroxidase-conjugated goat anti-rabbit antibody (Sigma, www.sigmaaldrich.com) was used as the secondary antibody at a 1:5,000 dilution in PBS-Tween, and blots were developed using ECL Western Blotting Detection Reagents (Amersham Pharmacia Biotech, www.apbiotech.com).

### VSV-GFP replication in embryonic termite cultures

Primary embryonic *C. formosanus* cells derived from approximately 230 eggs were infected for 1 h followed by five washes with 500 µl of TCCM to remove unbound input VSV-GFP. The fifth wash was collected to determine amount of input virus that remained in the cultures. Cell culture supernatants were collected sequentially (500 µl each), filtered through a 0.45 micron filter at 1, 24, 48, 72, and 96 h post-infection, and stored at -80°C. BHK-21 cell monolayers in 96-well tissue culture plates were incubated with triplicate ten-fold serial dilutions of collected supernatants to determine viral titers as plaque-forming units per ml (pfu/ml). Cells were fixed with a 10% formalin solution (Formade-Fresh, Fisher Scientific) and stained with crystal violet (1% (w/v) crystal violet in 70% ethanol) 5 days post-infection to quantify cytopathic effects (CPE).

### Infection of termite gut with recombinant VSV

*C. formosanus* workers that had been starved for 48 h to eliminate wood pulp in their guts were surface sterilized with 1 ml of the following solutions: 0.05% (w/v) NaHClO_4_ (x1), twice with dH_2_O, 70% (v/v) ethanol (x1), twice with dH_2_O , and twice with TCCM. Using microforceps, termites were dissected by pulling off the head and abdomen. Guts were removed from the head and abdomen and placed into wells (12 per well) of a 24-well tissue culture plate each containing 500 µl TCCM. Guts were inoculated with 1.5× 10^7^ FFU of VSV-GFP for 1 h and media was replaced with fresh TCCM. Infected guts were cultured at 27°C and remained viable for at least 48 h as evidenced by contraction. GFP expression was analyzed by fluorescence microscopy 24 and 48 h post-infection.

## Results

**Figure 1.**
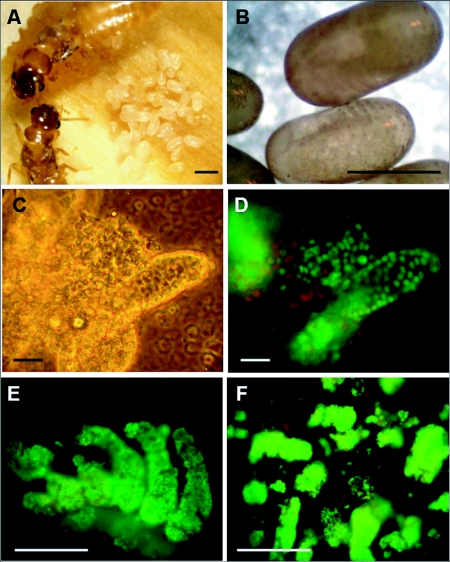
Formosan subterranean termite, *Coptotermes formosanus*, eggs and embryonic primary cultures. (A) adult workers caring for eggs. (B) Late stage eggs with developing embryos used for cell culture. (C) Bright field image of an embryonic primary culture. (D) Viability of primary embryonic cell culture. Nuclear green staining with SYBR14 indicates cells that are viable. Propidium iodide stains the nuclei of dead cells red. (E) Viability of cryopreserved embryonic primary culture immediately after thawing, stained with SYBR14 and propidium iodide. (F) Viability of the same cryopreserved culture 24 h later. Scale bars, 1 mm (A); 500 µm (B); 20 µm (C, D); 50 µm (E, F).

As there are no existing cell lines derived from termite tissues to facilitate the testing of gene transfer vectors, we first developed a method for establishing primary cell cultures derived from *C. formosanus* (Figure 1A, B) and *R. flavipes* embryos that allowed the reproducible maintenance of viable cultures for at least one week. Eggs containing advanced stage embryos were surface sterilized and gently homogenized. The resulting embryonic primary cell cultures were heterogeneous in morphology and included individual cells, cell clusters and embryo pieces in suspension (Figure 1C). To determine the viability of these cells, nucleic acid stains propidium iodide and SYBR 14 were added to the cultures. Propidium iodine is a membrane-impermeant dye that will stain the DNA of damaged or membrane-compromised cells, whereas SYBR 14 is a membrane-permeant dye that will stain both viable and damaged cells. With this assay, damaged cells stain red while intact, viable cells stain green (Garner et al. 1994). Figure 1D demonstrates a typical population of viable *C. formosanus* embryonic cells stained with SYBR 14 and propidium iodine. Similar cultures were obtained with *R. flavipes* cells (data not shown).

Due to the seasonality and limited year-round availability of termite eggs, whether these primary cultures derived from termite embryos could be cryopreserved was examined. Primary *C. formosanus* embryonic cultures were resuspended in cryopreservation media prior to storage in liquid nitrogen. After 24 h of cold storage, embryonic cell cultures were re-seeded in termite cell culture medium. The viability of these cultures was determined using the fluorescent dyes SYBR 14 and propidium iodine upon thawing and 24 h later (Figure 1E, F). A large proportion of embryonic cells remained viable with little marked difference between the viability of fresh cultures and cryopreserved cultures.

**Figure 2.**
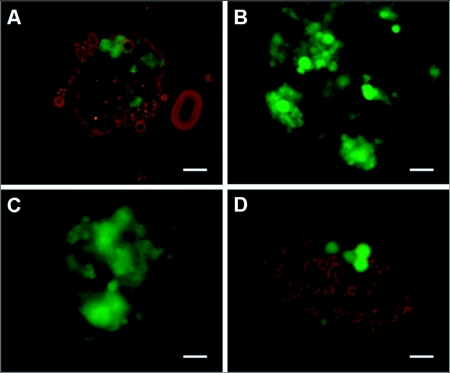
GFP expression in VSV-GFP infected termite embryonic primary cultures. (A) GFP expression in VSV-GFP infected *Coptotermes formosanus* embryonic cells at 24 h, (B) 48 h, and (C) 1 week post-infection. (D) GFP expression in VSV-GFP infected *Reticulitermes flavipes* embryonic cells at 48 h post-infection. Scale bars, 10 µm (A–D).

An infectious cDNA clone of VSV engineered to express the marker gene, green fluorescent protein (GFP), VSV-GFP, has been previously described (Cherry et al. 2005; Whelan et al. 1995; Whelan et al. 2000). To determine whether VSV-GFP could infect termite embryonic cultures and whether heterologous gene transfer could be achieved, primary cells derived from *C. formosanus* and *R. flavipes* embryos were treated with VSV-GFP. In *C. formosanus* cultures, 24 h post-infection, cytoplasmic GFP expression could be detected by fluorescence microscopy (Figure 2A). At 48 h, the level of protein expression increased (Figure 2B) and was maintained for at least 7 days (Figure 2C). The majority of cells expressed GFP by 48 hr. In *R. flavipes* cultures, similar results were obtained with a 48 h timepoint shown in Figure 2D. No green fluorescence was observed in non-infected control cultures (data not shown). These results are consistent with virus entry and heterologous gene expression.

**Figure 3.**
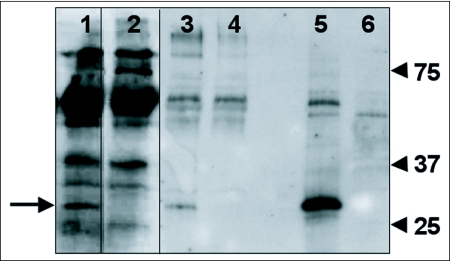
Anti-GFP immunoblots of VSV-GFP infected embryonic termite cells. Lane 1, VSV-GFP infected *Coptotermes formosanus* cells at 96 h post-infection. Lane 2, uninfected *C. formosanus* cells. Lane 3, VSV-GFP infected *Reticulitermes flavipes* cells at 48 h post-infection. Lane 4, uninfected *R. flavipes* cells. Lane 5, VSV-GFP infected BHK-21 cells. Lane 6, uninfected BHK-21 cells. Gels were run under the same conditions. Molecular size markers (kDa) are shown on the right. The GFP band is marked by an arrow on the left.

To confirm GFP expression in embryonic tissues, cultures were collected and a Western immunoblot assay was performed using an anti-GFP antibody with VSV-GFP infected and uninfected *C. formosanus* and *R. flavipes* cell lysates as well as positive control VSV-GFP infected baby hamster kidney (BHK-21) cells (Figure 3). A band was detected in the VSV-GFP infected *C. formosanus* (lane 1) and *R. flavipes* (lane 3) cell lysates that corresponded to the GFP band detected in the infected BHK control (lane 5). The immunoblot results corroborate the fluorescence microscopy results and confirm that VSV-GFP can infect both *C. formosanus* and *R. flavipes* embryonic primary cultures and express a heterologous protein, GFP.

**Figure 4.**
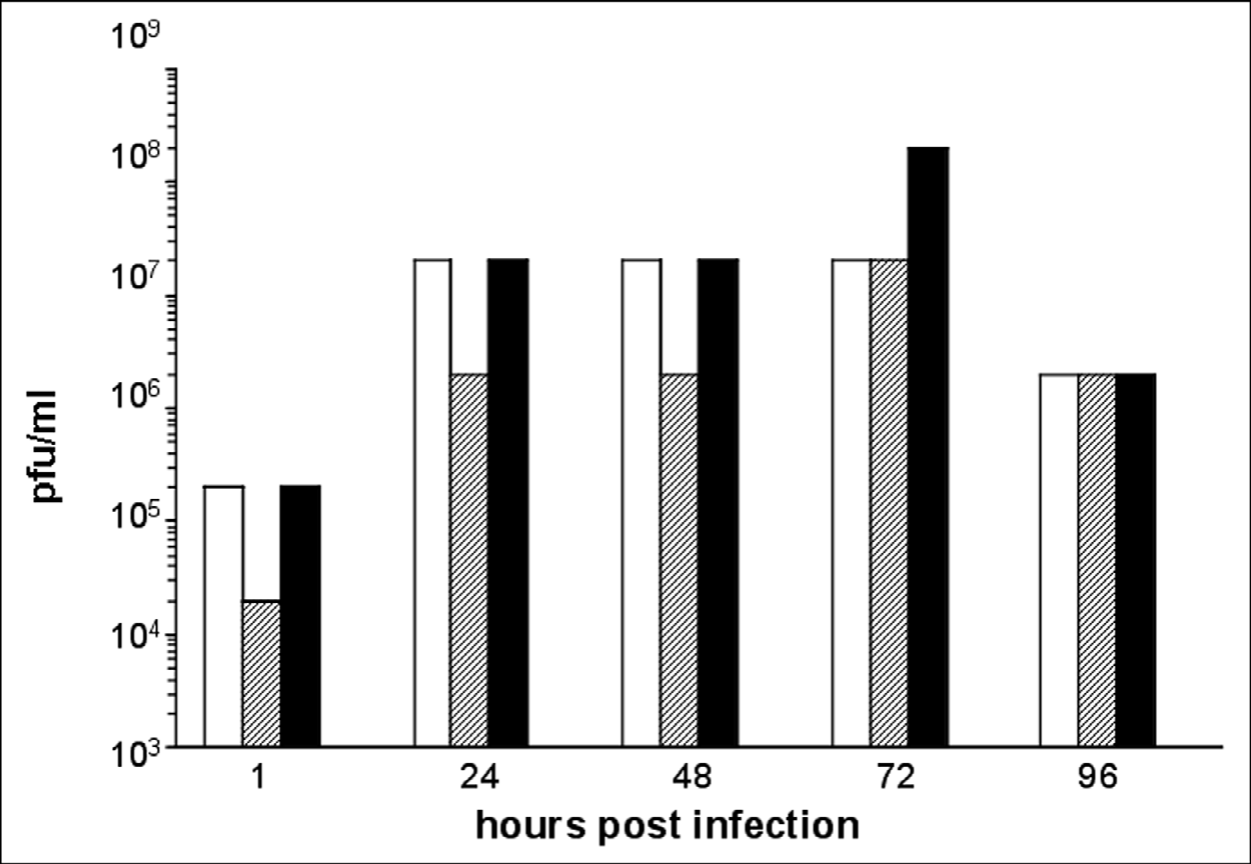
De *novo* replication of VSV-GFP in *Coptotermes formosanus* embryonic primary cultures. Supernatant of VSV-GFP infected termite cells collected at 1, 24, 48, 72, and 96 h post-infection was titered on BHK-21 cells in ten-fold triplicate serial dilutions. Titers were scored in pfu/ml as the highest dilution that yielded at least1 pfu.

To determine whether VSV could replicate and produce progeny virions in *C. formosanus* embryonic primary cells, supernatants from VSV-GFP infected termite cells were sequentially collected at several time points post-infection and added to BHK-21 cells in ten-fold serial dilutions. As VSV is stable at room temperature for several days, termite cells were washed five times with TCCM after infection to reduce the amount of input virus that remained in the cultures. At 24 h, viral titers increased two orders-of-magnitude above initial post-infection titers (Figure 4). The amount of virus collected in the next 24 hr period at 48 hr post-infection remained two orders-of-magnitude above initial titers. Viral titers remained the same as compared to 48 h post-infection or increased an additional order of magnitude 72 h post-infection. Viral levels remained above initial post-infection levels 96 h after infection . These findings are consistent with *de novo* virus replication in *C. formosanus* embryonic cells.

**Figure 5.**
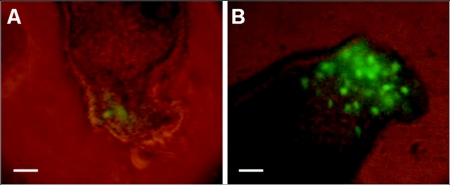
GFP expression in VSV-GFP infected termite gut. Anterior portion of *Coptotermes formosanus* gut infected with VSV-GFP express GFP at (A) 24 h and (B) 48 h. Scale bars, 100 µm (A, B).

To determine whether VSV-GFP could be used as a gene transfer vector in adult termite tissues, *C. formosanus* worker guts were isolated and cultured in TCCM and infected with VSV-GFP. Within 24 h, GFP expression could be detected by fluorescent microscopy in the anterior portion of the gut where it had been dissected from the head (Figure 5A). At 48 h, the level of gene expression increased and was maintained for as long as the gut cultures remained viable, for up to a week (Figure 5B). GFP expression only in the injured region of the gut is consistent with VSV entry.

## Discussion

In this study, a gene transfer method into fresh and cryopreserved cell cultures from embryos of *C. formosanus* and *R. flavipes* was developed using a recombinant VSV vector encoding GFP (Whelan et al. 2000; Cherry et al. 2005). VSV-GFP infection of primary cell cultures derived from *C. formosanus* and *R. flavipes* embryos as well as adult *C. formosanus* gut tissue resulted in foreign gene expression. In addition, infectious progeny virions were isolated from infected cells consistent with VSV replication in termite cells.

In explanted adult gut cultures, infection and transgene expression was limited to the proximal, torn end of the gut and not along the intact length of the gut or the distal end. This is consistent with the known tropism of VSV, which infects the basolateral side of polarized epithelial cells in culture and not the apical side (Fuller et al. 1984; Pfeiffer et al. 1985). In support of this, we were unable to show evidence of infection or transgene expression in adult termites that were fed VSV-soaked filter paper,or microinjected with VSV (although the microinjection experiments were compromised by the tendancy of injected termites to be eaten by their colony mates if housed together, or to stop feeding and show signs of rapidly deteriorating health if housed alone) (data not shown). We hypothesize that there may be immunological effects that prevent productive VSV infection in adults and adult tissue explants. Recently anti-viral RNAi activity was shown against VSV in *C. elegans* (Schott et al. 2005).

Other transgene related control strategies have involved expression of foreign genes in termite bacterial gut fauna. In one study, GFP expressing gut bacteria were introduced into *C. formosanus* as a shuttle system to deliver and spread foreign genes into bacterial symbiont populations of termite colonies (Husseneder and Grace 2005). They found that these transgenic indigenous gut bacteria were not cleared by the termites as compared to using a genetically-modified non-native bacterium such as *Eschenchia coli* (Husseneder et al. 2005). However, these strategies address only the genetics of the bacterial symbionts, but not the termites and thus do not allow the study of termite genes or gene targets.

During the course of these studies, we attempted to express heterologous genes in termite cells using a variety of other methods including electroporation, lipid-mediated transfection, particle bombardment, as well as other viral vectors with limited success. With regard to the viral vectors, we attempted to infect primary cultures derived from termite embryos with recombinant retroviral vectors derived from the parent viruses Moloney murine leukemia virus (MoMLV) and human immunodeficiency virus-1 (HIV-1) (Emi et al. 1991; Miller and Rosman 1989; Zhang et al. 2002). Upon entry into permissive cells these retroviral vectors stably integrate their genomes into the genome of infected host cells. Similar to VSV-GFP, both of these viral systems encoded GFP. In addition, both the HIV-1 and MoMLV virions had been pseudotyped with the glycoprotein of VSV (VSV-G). We hypothesized that VSV-G on the surface of the pseudotyped retroviruses would bind to its native receptor and allow entry into termite cells as had VSV. However, neither MoMLV nor HIV-1 vectors expressed transgenes in termite cells as measured by fluorescence microscopy. This result was not necessarily surprising for MoMLV, as this virus requires active cell division for integration (Miller et al. 1990). Though viable, embryonic termite cell cultures did not appear to be actively dividing by microscopic observation, as cell numbers did not appear to increase over time. HIV-1-based vectors have the ability to integrate into the genome of host cells in the absence of nuclear division (Lewis and Emerman 1994). It appears that factors required for a productive HIV-1 infection in termite cells are lacking. As VSV-G pseudotyped HIV-1 particles were used, this block is likely post-entry.

In contrast to retroviral vectors that require nuclear transport and integration, the replication of VSV is entirely cytoplasmic. The ability of another cytoplasmic virus, the positive-stranded RNA alphavirus Sindbis, to infect primary termite cell cultures was also tested. Similar Sindbis virus systems have been used to both express foreign proteins as well as RNAi for gene silencing in several mosquito and moth species (Foy et al. 2004; Adelman et al. 2001). Based on lack of observed green fluorescence in infected cell cultures, Sindbis virus vectors were unable to infect termite cells, indicating that the tropism of the recombinant Sindbis virus vector in termite cells is limited by the tropism of the parent virus (Nilkasson 1988). At this point, it cannot be ascertained whether this is due to a block in entry, replication, or transgene expression. In our hands, this Sindbis virus system successfully expressed transgenes in cell lines derived from other invertebrates (data not shown).

Based on this work, it is now possible to perform studies where candidate termite or heterologous genes are introduced and expressed in termite cells in culture in order to independently study their effects at a molecular and cellular level. This can include both the expression of proteins and RNA molecules. Recent work on *R. flavipes* has identified genes that are differentially regulated in immature and adult reproductive castes using macroarray-based genomic techniques (Scharf et al. 2003; Scharf et al. 2005). The effects of these genes and other genes could be independently studied in termite primary cell culture using recombinant VSV vectors. Examples of other exogenous genes that might be expressed with this system include immortalizing proteins or RNA molecules, such as cell cycle regulators, growth factor or other signaling molecules, or telomere extension factors that might be used to generate permanent termite cell lines. Potential biocontrol genes, including bacterial, fungal, or viral toxin genes, signaling molecules, immune modulators, or developmental control molecules could also be used to design transgenic VSV vectors with high level pathogenicity against termites.

To our knowledge, the present work is the first evidence of *ex vivo* cell culture of termite embryonic cells, their cryopreservation, and gene transfer in these cells. The availability of viable embryonic termite cell cultures and the ability to introduce genes into termite tissues permits the study of the molecular details of termite cell biology and will facilitate the study of important genes and key regulatory pathways. There does not appear to be a single gene transfer technology that is robust or plastic enough to perform efficiently in all invertebrate organisms. We have found that VSV vectors can efficiently enter and mediate expression of foreign transgenes in termite cells. The results of this work provide additional tools for the genetic study of termites, and alternatives for the molecular study of other important invertebrate organisms for which gene transfer technologies have been lacking to date.

## Abbreviations

FFU -: focus forming units
GFP -: green fluorescent protein
TCCM -: termite cell culture medium
VSV -: negative-stranded RNA rhabdovirus of Vesiculovirus

## References

[bibr01] Adelman ZN, Blair CD, Carlson JO, Beaty BJ, Olson KE (2001). Sindbis virus-induced silencing of dengue viruses in mosquitoes.. *Insect Molecular Biology*.

[bibr02] Cherry S, Doukas T, Armknecht S, Whelan S, Wang H, Sarnow P, Perrimon N (2005). Genome-wide RNAi screen reveals a specific sensitivity of IRES-containing RNA viruses to host translation inhibition.. *Genes and Development*.

[bibr03] Emi NT, Friedman T, Yee JK (1991). Pseudotype formation of murine leukaemia virus with the G protein of vesicular stomatitis virus.. *Journal of Virology*.

[bibr04] Foy BD, Myles KM, Pierro DJ, Sanchez-Vargas I, Uhlirova M, Jindra M, Beaty BJ, Olson KE (2004). Development of a new Sindbis virus transducing system and its characterization in three Culicine mosquitoes and two Lepidopteran species.. *Insect Molecular Biology*.

[bibr05] Fuller S, von Bonsdorff CH, Simons K (1984). Vesicular stomatitis virus infects and matures only through the basolateral surface of the polarized epithelial cell line, MDCK.. *Cell*.

[bibr06] Garner DL, Johnson LA, Yue ST, Roth BL, Haugland RP (1994). Dual DNA staining assessment of bovine sperm viability using SYBR 14 and propidium iodide.. *Journal of Andrology*.

[bibr07] Gay FJ, Krishna K, Weesner FM (1969). Species introduced by man.. *Biology of Termites* 1.

[bibr08] Gillies S, Stollar V (1980). The production of high yields of infectious vesicular stomatitis virus in *Aedes albopictus* cells and comparison with replication in BHK-21 cells.. *Virology*.

[bibr09] Husseneder C, Grace JK, Oishi DE (2005). Use of genetically engineered *Escherichia coli* to monitor ingestion, loss, and transfer of bacteria in termites.. *Current Microbiology*.

[bibr10] Husseneder C, Grace JK (2005). Genetically engineered termite gut bacteria (*Enterobacter cloacae*) deliver and spread foreign genes in termite colonies.. *Applied Microbiology and Biotechnology*.

[bibr11] Kofoid CA (1946). *Termites and Termite Control*2nd edition..

[bibr12] Lax AR, Osbrink WLA (2003). United States Department of Agriculture — Agriculture Research Service research on targeted management of the Formosan subterranean termite *Coptotermes formosanus* Shiraki (Isoptera: Rhinotermitidae).. *Pest Management Science*.

[bibr13] Lewis PF, Emerman M (1994). Passage through mitosis is required for oncoretroviruses but not for human immunodeficiency virus.. *Journal of Virology*.

[bibr14] Matlin KS, Reggio H, Helenius A, Simons K (1982). Pathway of vesicular stomatitis virus entry leading to infection.. *Journal of Molecular Biology*.

[bibr15] Miller DG, Adam MA, Miller AD (1990). Gene transfer by retrovirus vectors occurs only in cells that are actively replicating at the time of infection.. *Molecular and Cellular Biology*.

[bibr16] Miller DA, Rosman GJ (1989). Improved retroviral vectors for gene transfer and expression.. *Biotechniques*.

[bibr17] Nilkasson B, Monath TP (1988). Sindbis and Sindbis-like viruses.. *The Arboviruses: Epidemics and Ecology* 4.

[bibr18] Pfeiffer S, Fuller SD, Simons K (1985). Intracellular sorting and basolateral appearance of the G protein of vesicular stomatitis virus in Madin-Darby canine kidney cells.. *Journal of Cell Biology*.

[bibr19] Raina A, Park YI, Florane C (2003). Behavior and reproductive biology of the primary reproductives of the Formosan subterranean termite (Isoptera: Rhinotermitidae).. *Sociobiology*.

[bibr20] Robinson CD, Lourido S, Whelan SP, Dudycha JL, Lynch M, Isern S (2006). Viral transgenesis of embryonic cell cultures from the freshwater microcrustacean *Daphnia*.. *Journal of Experimental Zoology A Comparative and Experimental Biology*.

[bibr21] Scharf ME, Wu-Scharf D, Zhou X, Pittendrigh BR, Bennett GW (2005). Gene expression profiles among immature and adult reproductive castes of the termite *Reticulitemes flavipes*.. *Insect Molecular Biology*.

[bibr22] Scharf ME, Wu-Scharf D, Pittendrigh BR, Bennett GW (2003). Caste-and development-associated gene expression in the lower termite.. *Genome Biology*.

[bibr23] Schott DH, Cureton DK, Whelan SP, Hunter CP (2005). An antiviral role for the RNA interference machinery in *Caenorhabditis elegans*.. *Proceedings of the National Academy of Science USA*.

[bibr24] Shope RE, Tesh RB, Wagner RR (1987). The ecology of rhabdoviruses that infect vertebrates.. *The Rhabdoviruses*.

[bibr25] Tesh RB, Chaniotis BN, Johnson KM (1972). Vesicular stomatitis virus (Indiana serotype): transovarial transmission by phlebotomine sandflies.. *Science*.

[bibr26] Tesh RB, Peralta PH, Johnson KM (1970). Ecologic studies of vesicular stomatitis virus II. Results of experimental infection in Panamanian wild animals.. *American Journal of Epidemiology*.

[bibr27] Whelan SP, Ball LA, Barr JN, Wertz GW (1995). Efficient recovery of infectious vesicular stomatitis virus entirely from cDNA clones.. *Proceedings of the National Academy of Science USA*.

[bibr28] Whelan SP, Barr JN, Wertz GW (2000). Identification of a minimal size requirement for termination of vesicular stomatitis virus mRNA: implications for the mechanism of transcription.. *Journal of Virology*.

[bibr29] Zhang X-Y, La Russa VF, Bao L, Kolls J, Schwarzenberger P, Reiser J (2002). Lentiviral vectors for sustained transgene expression in human bone marrow-derived stromal cells.. *Molecular Therapy*.

